# 3-[4-(1*H*-Indol-3-yl)-1,3-thiazol-2-yl]-1*H*-pyrrolo[2,3-*b*]pyridines, Nortopsentin Analogues with Antiproliferative Activity

**DOI:** 10.3390/md13041901

**Published:** 2015-04-03

**Authors:** Barbara Parrino, Anna Carbone, Gloria Di Vita, Cristina Ciancimino, Alessandro Attanzio, Virginia Spanò, Alessandra Montalbano, Paola Barraja, Luisa Tesoriere, Maria Antonia Livrea, Patrizia Diana, Girolamo Cirrincione

**Affiliations:** Dipartimento di Scienze e Tecnologie Biologiche Chimiche e Farmaceutiche, STEBICEF, via Archirafi 32, 90123 Palermo, Italy; E-Mails: barbara.parrino@unipa.it (B.P.); anna.carbone@unipa.it (A.C.); gloria.divita@unipa.it (G.D.); cristina.ciancimino@unipa.it (C.C.); alessandro.attanzio@unipa.it (A.A.); virginia.spano@unipa.it (V.S.); alessandra.montalbano@unipa.it (A.M.); paola.barraja@unipa.it (P.B.); luisa.tesoriere@unipa.it (L.T.); maria.livrea@unipa.it (M.A.L.); patrizia.diana@unipa.it (P.D.)

**Keywords:** marine alkaloids, indolyl alkaloids, nortopsentin analogues, 3-[4-(1*H*-Indol-3-yl)-1,3-thiazol-2-yl]-1*H*-pyrrolo[2,3-*b*]pyridines, antiproliferative activity

## Abstract

A new series of nortopsentin analogues, in which the imidazole ring of the natural product was replaced by thiazole and the indole unit bound to position 2 of the thiazole ring was substituted by a 7-azaindole moiety, was efficiently synthesized. Two of the new nortopsentin analogues showed good antiproliferative effect against the totality of the NCI full panel of human tumor cell lines (~60) having GI_50_ values ranging from low micromolar to nanomolar level. The mechanism of the antiproliferative effect of these derivatives, investigated on human hepatoma HepG2 cells, was pro-apoptotic, being associated with externalization of plasma membrane phosphatidylserine and mitochondrial dysfunction. Moreover, the compounds induced a concentration-dependent accumulation of cells in the subG0/G1phase, while confined viable cells in G2/M phase.

## 1. Introduction

New natural or synthetic heterocyclic scaffolds have been recently identified and developed as possible anticancer agents [1,2,3,4,5,6,7,8,9,10,11,12,13,14,15,16,17,18,19]. Among the various natural sources, viz plants, microbes, animals, and marine organisms, the latter are increasingly contributing a large number of biologically active alkaloids. An important class of deep-sea sponge metabolites with remarkable biological activities, such as anti-inflammatory, antimicrobial, antiviral, and antitumor, is constituted by bis-indolyl alkaloids, characterized by two indole units connected, through their position 3, by a spacer [20,21,22,23].

**Chart 1:**
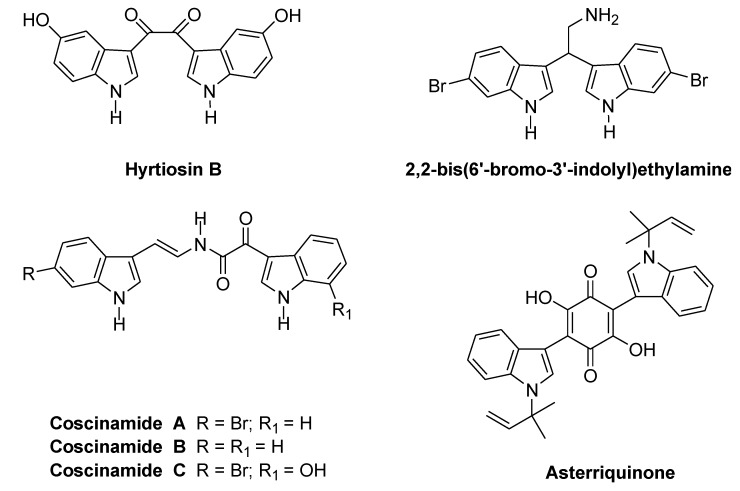
bis-Indolyl alkaloids with acyclic and carbocyclic spacers.

The bis-indolyl alkaloid spacer can have several structural features such as an acyclic chain or a carbocyclic ring. Thus, hyrtiosin B, isolated from *Hyrtios erecta*, 2,2-bis(6′-bromo-3′-indolyl)ethylamine, isolated from the tunicate *Didemnum candidum* and Coscinamides A–C, isolated from deep marine sponge *Coscinoderma*, showed antiproliferative or anti-HIV activity, as well as asterriquinone, isolated from *Aspergillus fungi*, and bearing a six-membered carbocyclic symmetrical structure (Chart 1) [24,25,26,27].

**Chart 2:**
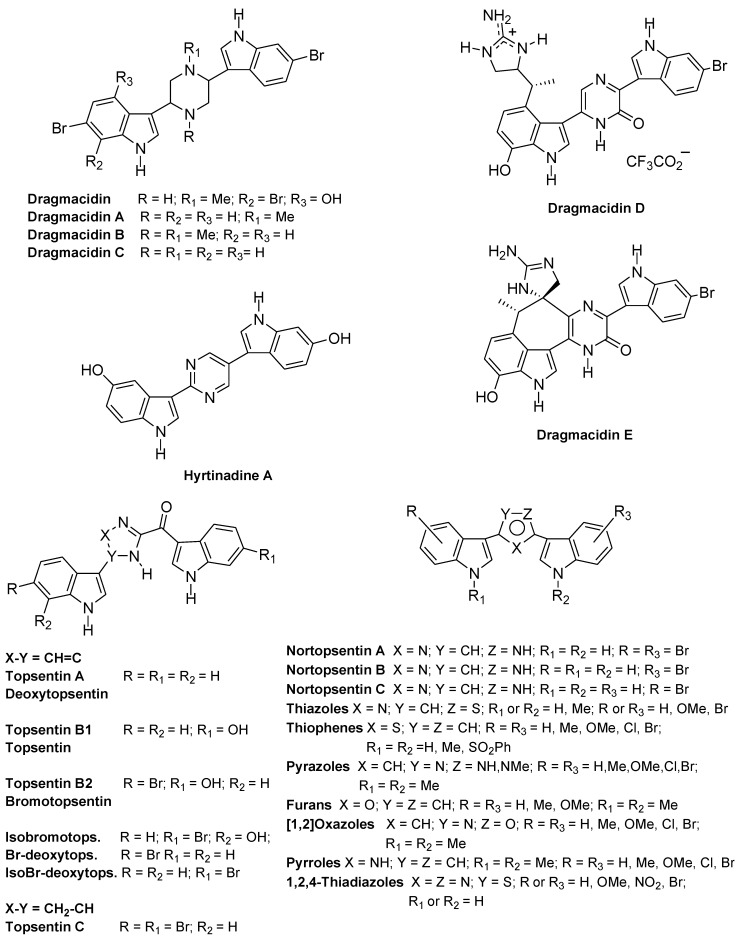
bis-Indolyl alkaloids and analogues with heterocyclic spacers.

The spacer can also be constituted by differently sized heterocycles. Thus, dragmacidin and dragmacidins A−E, isolated from a large number of deep water sponges such as *Dragmacidon*, *Halicortex*, *Spongosorites*, *Hexadella* and the tunicate *Didemnum candidum*, exhibit the saturated six-membered heterocyclic link piperazine or a pyrazinone spacer, and showed several biological properties (Chart 2) [25,28,29,30,31].

More recently, hyrtinadine A, isolated from the marine sponge *Hyrtios* and bearing a pyrimidine ring as a spacer, exhibited *in vitro* cytotoxicity [32].

Topsentins A, B1 and B2, which were isolated from Mediterranean sponge *Topsentia genitrix*, bear a 2-acyl imidazole spacer and showed antitumor and antiviral activities [33]. Later, from *Spongosorites* sp. and from *Hexadella* sp. were obtained the same natural products, which were named deoxy-topsentin, topsentin, and bromotopsentin, respectively [34,35]. More recently, topsentin C, isobromotopsentin, bromodeoxytopsentin, and its isomer isobromodeoxytopsentin, were isolated from *Hexadella* sp., *Spongosorites* sp and *Spongosorites genitrix*, respectively [29,36,37,38].

Nortopsentins A−C, isolated from *Spongosorites ruetzleri*, have a 2,4-disubstituted imidazole ring as a spacer and showed *in vitro* cytotoxicity against P388 cells (IC_50_, 4.5–20.7 μM). Methylation of the two-indole nitrogen atoms produced a significant improvement in cytotoxicity (IC_50_, 0.64–1.70 μM) [39].

Since marine organisms allow the isolation of very small amount of the biologically active substances from the natural material, several total synthesis of nortopsentins were proposed [40,41,42,43]. Moreover, due to the considerable activities shown, indolyl alkaloids have attracted remarkable attention by researchers becoming an interesting field in medicinal chemistry. Thus, several dragmacidin analogues bearing six membered heterocycles, such as pyridine, pyrazine, pyrazinone and pyrimidine, as spacer and showing antiproliferative activity were synthesized [44,45,46,47].

Also, nortopsentins were considered lead compounds and several reports dealt with the synthesis and the evaluation of the antiproliferative activity of nortopsentin analogues bearing five-membered heterocycles, which replaced the imidazole ring of the natural product, such as bis-indolyl-thiophenes [48], -pyrazoles [49], -furans [50], -isoxazoles [50], -pyrroles [51], and -1,2,4-thiadiazoles [52]. Most of these analogues showed antiproliferative activity often reaching GI_50_ values at submicromolar level.

Beside the eterocyclic spacer, the structural manipulation of the natural nortopsentins, was extended to one or both indole units and produced 3-[(2-indolyl)-5-phenyl]pyridine and phenylthiazolyl-7-azaindole derivatives, which showed antiproliferative activity and inhibited CDK1 [53,54].

**Chart 3:**
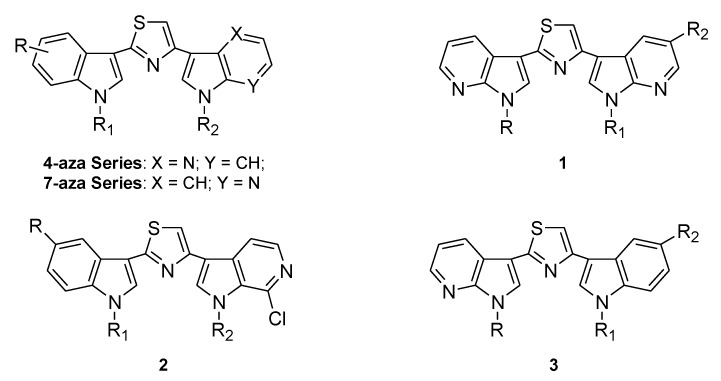
Nortopsentin aza-analogues.

More recently, due to the good results obtained by the aza-substitution of the indole moiety, 3-[2-(1*H*-indol-3-yl)-1,3-thiazol-4-yl]-1*H*-4-azaindole derivatives were synthesized and tested against breast cancer, prostate cancer, pancreatic carcinoma and peritoneal mesothelioma and showed cytotoxic activity, inhibited CDK1, and increased caspase-9 and caspase-3 activity (Chart 3) [55].

Contemporaneously, 3-[2-(1*H*-indol-3-yl)-1,3-thiazol-4-yl]-1*H*-7-azaindoles were synthesized and tested against the NCI full panel of human cancer cell lines and STO and MesoII cells, derived from human diffuse malignant peritoneal mesothelioma (DMPM). The most active compounds, which also act as CDK1 inhibitors, administrated with paclitaxel produced a synergistic cytotoxic effect. In the mouse model, intraperitoneal (i.p.) administration of active derivatives was effective, resulting in a significant tumor volume inhibition of DMPM xenografts (range, 58%–75%) at well-tolerated doses, and two complete responses were observed in each treatment group [56].

Lately, two new series of nortopsentin analogues, **1** and **2**, were efficiently synthesized and inhibited the growth of HCT-116 colorectal cancer cells at low micromolar concentrations, whereas they did not affect the viability of normal-like intestinal cells. A compound of the series **1** induced apoptosis. Instead, a derivative of the series **2** at low concentrations (GI_30_) induced morphological changes characteristic of autophagic death with massive formation of cytoplasmic acid vacuoles without apparent loss of nuclear material, and with arrest of cell cycle at the G1 phase, whereas higher concentrations (GI_70_) induced apoptosis with arrest of cell cycle at the G1 phase [57].

In this paper, continuing our studies on indole and related azaindole systems [58,59,60,61,62,63,64], we report the synthesis of derivatives of type **3**, nortopsentin analogues, in which indole and 7-azaindole units of the preceding very active 7-aza series were switched. We also report the antiproliferative activity of these new nortopsentin analogues and studies directed to elucidate their mode of action.

## 2. Results and Discussion

### 2.1. Chemistry

**Scheme 1:**
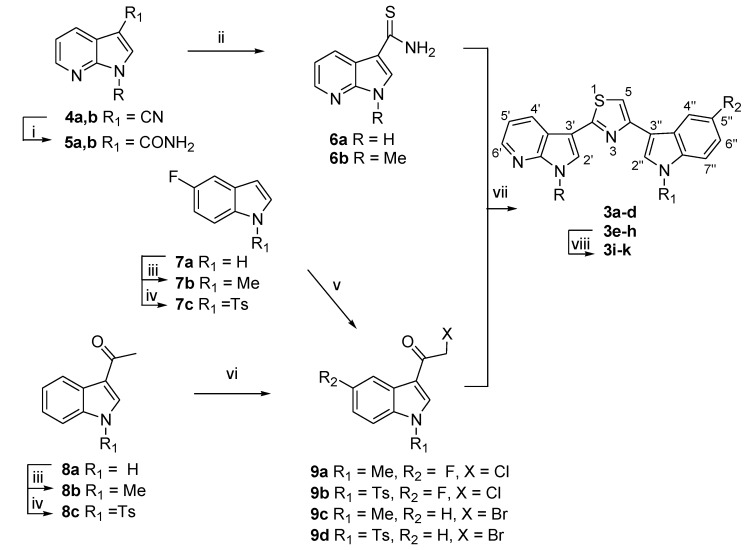
Synthesis of substituted 3-[4-(1*H*-indol-3-yl)-1,3-thiazol-2-yl]-1*H*-pyrrolo[2,3-*b*] pyridines **3a**–**k**. Reagents: (i) (a) H_2_SO_4_, 25 °C, 15–60 min; (b) NaOH, 95%–99%; (ii) Lawesson’s reagent, THF, reflux, 30 min, 88%–99%; (iii) (a) *t*-BuOK, toluene, tris[2-(2-methoxyethoxy)ethyl]amine (TDA-1), 25 °C, 5–8 h; (b) MeI, 25 °C, 1–2 h, 80%–98%; (iv) (a) NaH, THF, 0 °C–25 °C, 1–24 h; (b) *p*-TsCl, 25 °C, 4–24 h, 90%–96%; (v) AlCl_3_, CH_2_Cl_2_, ClCOCH_2_Cl, 1 h, 50%–55%; (vi) Br_2_, MeOH, reflux, 2 h, 40%, 70%; (vii) EtOH, reflux, 30 min, 30%–97%; (viii) KOH, EtOH, reflux, 1–2 h, 40%–98%.

Substituted 3-[4-(1*H*-indol-3-yl)-1,3-thiazol-2-yl]-1*H*-pyrrolo[2,3-*b*]pyridines of type **3** (Scheme 1, Table 1) were obtained by Hantzsch reaction between pyrrolo[2,3-*b*]pyridine-carbothioamides **6a**,**b** and 3-haloacetyl compounds **9a**–**d**.

The key intermediates **6a**,**b** were obtained from the corresponding pyrrolo[2,3-*b*]pyridine-3-carbonitriles **4a**,**b** through the formation of carboxamides **5a**,**b**, as previously reported [57].

*N*-protected 5-fluoroindoles **7b**,**c** and 1-(1*H*-indol-3-yl)ethanones **8b**,**c** were synthesized, in good yields (80%–98%), from commercially available 5-fluoroindole **7a** or 1-(1*H*-indol-3-yl)ethanone **8a** by methylation [54], or reaction with tosyl chloride using sodium hydride (NaH) as the base in tetrahydrofuran (THF).

3-Haloacetyl indoles **9a**–**d** were prepared from the corresponding indoles **7b**,**c** or 1-(1*H*-indol-3-yl)ethanones **8b**,**c**. In particular, 3-chloroacetyl derivatives **9a**,**b** were obtained by reaction of the corresponding *N*-methyl or *N*-tosyl 5-fluoroindole **7b**,**c** with chloroacetyl chloride (ClCOCH_2_Cl) in the presence of aluminum chloride (AlCl_3_) in dichloromethane (CH_2_Cl_2_) in moderate yields (50%–55%); while 3-bromoacetyl compounds **9c**,**d** were synthesized (40%–70%) from the corresponding *N*-methyl or *N*-tosyl 1-(1*H*-indol-3-yl)ethanone **8b**,**c** using bromine in refluxing methanol (MeOH).

Reaction between pyrrolo[2,3-*b*]pyridine-carbothioamides **6a**,**b** and 3-haloacetyl compounds **9a**–**d** in ethanol under reflux afforded the desired 3-[4-(1*H*-indol-3-yl)-1,3-thiazol-2-yl]-1*H*-pyrrolo[2,3-*b*]pyridines **3a**–**h** in moderate to excellent yields (30%–97%) (Table 1).

The subsequent deprotection of *N*-tosyl derivatives **3e**–**h** using potassium hydroxide in refluxing ethanol gave the corresponding compounds **3i**–**k** (40%–98%).

**Table 1:** Substituted 3-[4-(1*H*-indol-3-yl)-1,3-thiazol-2-yl]-1*H*-pyrrolo[2,3-*b*]pyridine **3a**–**k**.

Compound	R	R_1_	R_2_	Yield%	Compound	R	R_1_	R_2_	Yield%
**3a**	H	Me	F	97	**3g**	H	Ts	H	90
**3b**	Me	Me	F	30	**3h**	Me	Ts	H	50
**3c**	H	Me	H	60	**3i**	H	H	F	98
**3d**	Me	Me	H	60	**3l**	Me	H	F	40
**3e**	H	Ts	F	97	**3j**	H	H	H	98
**3f**	Me	Ts	F	40	**3k**	Me	H	H	98

### 2.2. Biology

All the synthesized thiazoles **3a**–**k** were evaluated by the National Cancer Institute (Bethesda MD) for cytotoxicity against the NCI-60 cell line panel using standard protocols [65]. Initially, the selected derivatives **3c**,**d**,**g**,**h**,**j**,**k** were tested at a single dose (10^−5^ M) on the full panel of approximately 60 human tumor cell lines derived from 9 human cancer cell types, that have been grouped in disease sub-panels including leukemia, non-small-cell lung, colon, central nervous system (CNS), melanoma, ovarian, renal, prostate and breast cancers (data not shown). Compounds **3d** and **3k** were further selected for full evaluation at five concentration levels (10^−4^–10^−8^ M).

The antitumor activity of compounds **3d** and **3k** was given by three parameters for each cell line: GI_50_ (the molar concentration of the compound that inhibits 50% net cell growth), TGI (the molar concentration of the compound leading to total inhibition of net cell growth), and LC_50_ (the molar concentration of the compound that induces 50% net cell death). The average values of mean graph midpoint (MG_MID) were calculated for each of these parameters.

An evaluation of the data reported in Table 2 pointed out that compounds **3d** and **3k** exhibited antiproliferative activity against all the human cell lines at GI_50_ values from micromolar to nanomolar (13.0–0.03 and 14.2–0.04 µM, respectively).

**Table 2:** *In vitro* inhibition of cancer cell line growth by indolyl-thiazolyl-pyrrolopyridines **3d**, **3k** ^a^.

Cell Lines	GI_50_ (µM)		Cell Lines	GI_50_ (µM)		Cell Lines	GI_50_ (µM)	
	3d	3k		3d	3k		3d	3k
**Leukemia**			**CNS Cancer**			**Renal Cancer**		
CCRF-CEM	0.40	0.43	SF-268	2.44	0.94	786-0	0.97	0.72
HL-60(TB)	0.29	0.32	SF-295	0.24	0.31	A498	0.50	0.37
K-562	0.07	0.12	SF-539	0.18	0.25	ACHN	0.72	0.51
MOLT-4	0.67	0.66	SNB-19	0.63	0.64	CAKI-1	0.38	0.44
RPMI-8226	0.45	0.65	SNB-75	0.16	0.14	RXF393	0.33	0.52
SR	0.06	0.14	U251	0.41	0.43	SN12C	12.1	5.48
						TK-10	0.60	0.99
						UO-31	0.90	0.69
**Non-Small Cell Lung Cancer**			**Melanoma**			**Prostate Cancer**		
A549/ATCC	0.56	0.71	LOX IMVI	0.54	0.51	PC-3	0.46	0.56
EKVK	0.91	0.91	MALME-3M	1.04	0.74	DU-145	0.39	0.56
HOP-62	0.81	0.93	M14	0.27	0.28			
HOP-92	0.35	0.31	MDA-MB-435	0.03	0.04			
NCI-H226	0.74	0.65	SK-MEL-2	0.58	0.41			
NCI-H23	0.72	0.67	SK-MEL-28	1.20	0.76			
NCI-H322M	0.76	ND ^b^	SK-MEL-5	0.24	0.27			
NCI-H460	0.30	0.37	UACC-257	13.0	14.2			
NCI-H522	0.04	0.05	UACC-62	0.30	0.42			
**Colon Cancer**			**Ovarian Cancer**			**Breast Cancer**		
COLO-205	0.19	0.19	IGROV1	1.33	1.18	MCF7	0.33	0.37
HCC-2998	1.13	1.18	OVCAR-3	0.10	0.25	MDA-MB-231/ATCC	2.82	1.29
HCT-116	0.37	0.39	OVCAR-4	7.22	0.52	HS 578T	0.59	0.66
HCT-15	0.17	0.29	OVCAR-5	1.41	2.39	BT-549	0.47	0.44
HT29	0.25	0.19	OVCAR-8	0.66	0.82	MDA-MB-468	0.28	0.23
KM12	0.21	0.40	NCI/ADR-RES	0.09	0.15			
SW-620	0.22	0.30	SK-OV-3	0.57	0.52			

^a^ Data obtained from NCI’s *in vitro* disease-oriented tumor cells screen; ^b^ ND = not determined.

Indolyl-thiazolyl-pyrrolopyridine derivatives **3d** and **3k** were selective with respect to the leukemia cancer subpanel having all the subpanel cell lines GI_50_ in the range 0.06–0.67 μM and 0.12–0.66 μM, respectively. In particular, the most sensitive cell lines were K-562 (GI_50_ 0.07 μM and 0.12 μM, respectively), and SR (GI_50_ 0.06 μM and 0.14 μM, respectively). Derivatives **3d** and **3k** were also particularly sensitive against NCI-H522 (GI_50_ 0.04 μM and 0.05 μM, respectively) of non-small cell lung cancer, NCI/ADR-RES (GI_50_ 0.09 μM and 0.15 μM, respectively) of ovarian cancer, and MDA-MB-435 (GI_50_ 0.03 μM and 0.04 μM, respectively) of melanoma subpanel.

Cell growth inhibitory activity of **3d** and **3k** was also investigated on human HepG2 hepatocarcinoma.

**Figure 1:**
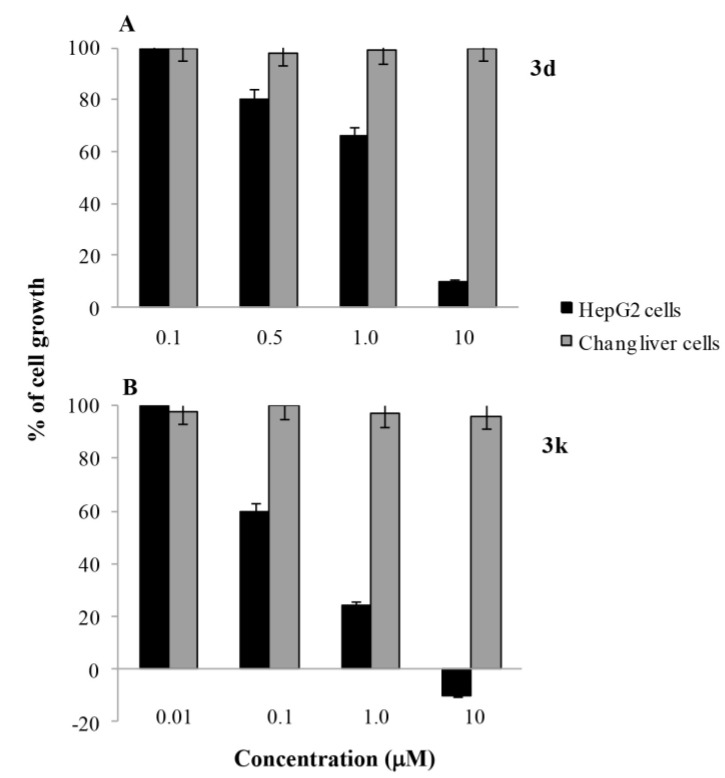
Effect **3d** (**A**) and **3k** (**B**) on the growth of HepG2 (black bars) or Chang cells (grey bars) as assessed by 3-(4,5-dimethylthiazol-2-yl)-2,5-diphenyltetrazolium bromide (MTT). Cell monolayers were incubated for 24 h in the absence (control) or in the presence of the compounds at the indicated concentrations and cell growth was assessed by MTT test as reported Methods. Results are indicated as the percentage of viable cells with respect to untreated controls. Values are the mean ± SD of three separate experiments carried out in triplicate.

Cell growth inhibitory activity of compounds **3d** and **3k** was also investigated on human HepG2 hepatocarcinoma cells, a cell line not included in the NCI panel and of interest in the drug discovery because provided with a very active microsomal system for detoxification of xenobiotics. Monolayer cultures treated for 72 h with 0.1–10 μM concentrations of the compounds were examined by 3-(4,5-dimethylthiazol-2-yl)-2,5-diphenyltetrazolium bromide (MTT) assay for cell growth. As shown in Figure 1, both the compounds inhibited the HepG2 cells growth in dose-dependent manner. Based on the dose response curve, the GI_50_ values were 1.69 µM) and 0.21 µM for **3d** and **3k**, respectively. Under identical conditions, both the nortopsentin analogues did not substantially impaired viability of normal immortalized human liver cells (Chang), suggesting high selectivity towards tumor cells.

**Figure 2:**
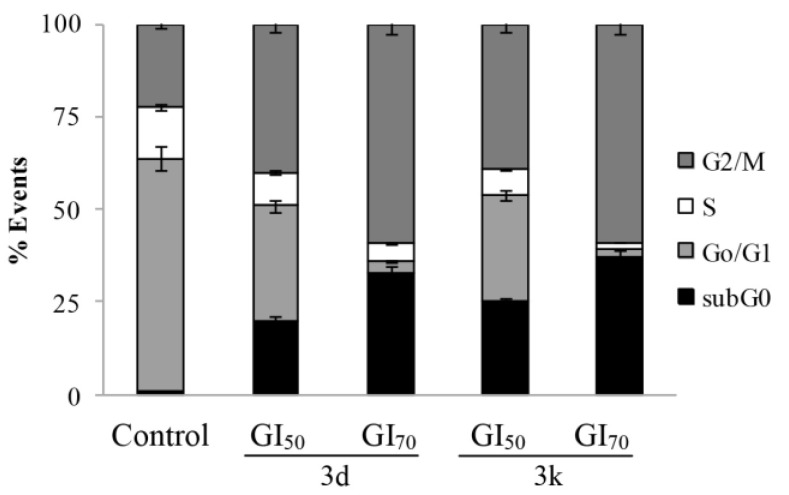
Effect of **3d** and **3k** on the cell cycle distribution of human hepatoma HepG2 cells. Flow cytometric analysis of propidium iodide (PI)-stained cells, as determined by flow cytometry after 24 h treatment with the compounds or vehicle alone (control). The percentage of cells in the different phases of the cycle was calculated by Expo32 software. Values are the mean ± SD of three separate experiments in triplicate.

The distribution of HepG2 cells in the cell cycle phases after 24 h treatment with the two norptopsentin analogues, was assessed by flow cytometric analysis after staining of DNA with PI. Both compounds **3d** and **3k** caused a significant dose-dependent decrease in the percentage of cells in the G0/G1 and S phases, accompanied by a concomitant percentage increase of cells in the G2/M phase, and appearance of a subG1-cell population (Figure 2).

**Figure 3:**
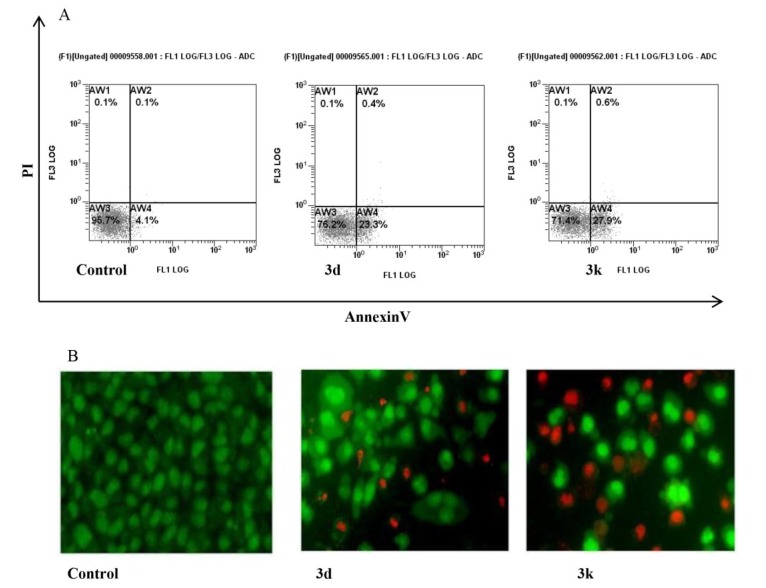
Apoptosis induced by **3d** an **3k** nortopsentin analogues in HepG2 cells. (**A**) Percentage of Annexin V/propidium iodide (PI) double-stained cells, as determined by flow cytometry; (**B**) Fluorescence micrographs of ethidium bromide/acridine orange double-stained cells. Control, cells treated with vehicle; **3d** and **3k**, cells treated for 24 h with either Nortopsentin derivative at GI_50_ concentration. (**A**) Representative images of three experiments with comparable results; (**B**) Representative images, in 200 × magnification, of three experiments with comparable results.

Apoptosis induction by **3d** and **3k** in HepG2 cells was investigated by externalization of plasma membrane phosphatidylserine and changes of mitochondrial transmembrane potential. Flow cytometry analysis of Annexin V-FITC/PI-stained cells after 24 h treatment with the Norptopsentin analogues, assayed at their relevant GI_50_ values, indicated a high percentage of cells in early apoptosis, with externalized phosphatidylserine (Figure 3A).

To confirm apoptotic mechanism of cytotoxicity of the nortopsentin analogues, we carried out morphological evaluation of HepG2 cells using AO and EB double staining. After 24 h of treatment with **3d** or **3k** at GI_50_ concentration, fluorescent microscopy revealed the appearance of cells containing bright green patches in the nuclei as a consequence of chromatin condensation and nuclear fragmentation, which are typical features of apoptosis. Moreover, fluorescing orange cells owing to increase of cell permeability to ethidium bromide, cell shrinkage and nuclear fragmentation were also evident as cells in late apoptosis (Figure 3B). Taken together, these findings provided strong evidence that the synthesized nortopsentin analogues induced apoptosis in HepG2 cells.

**Figure 4:**
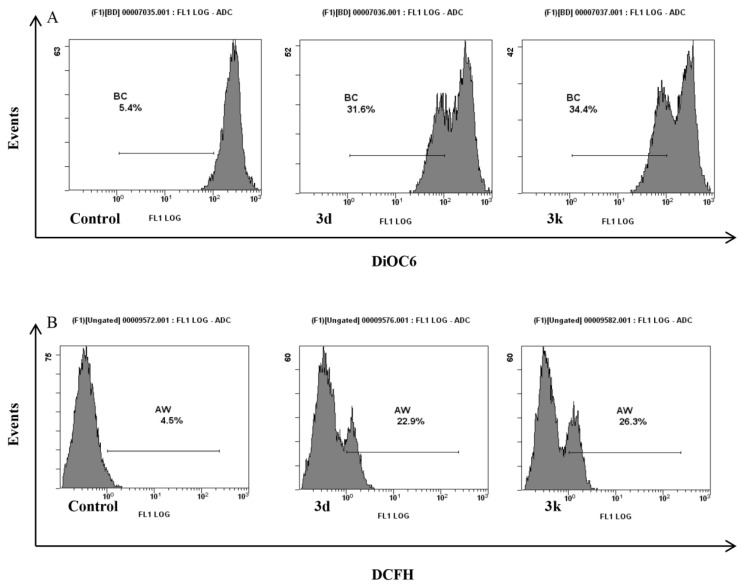
Mitochondrial dysfunction induced by **3d** and **3k** in HepG2 cells. (**A**) Fluorescence intensity of 3,30-dihexyloxacarbocyanine iodide-treated cells, as determined by flow cytometry; (**B**) Fluorescence intensity of 2′,7′-dichlorofluorescin diacetate-stained cells, as determined by flow cytometry. Control, cells treated with vehicle; **3d** and **3k**, cells treated for 24 h with either Nortopsentin derivative at GI_50_ concentration. Representative images of three experiments with comparable results.

Involvement of mitochondria in apoptosis induced in HepG2 cells by the synthesized nortopsentin analogues, was assessed. Loss of mitochondrial trans-membrane potential was indicated by decreased mitochondrial 3,30-dihexyloxacarbocyanine iodide-red fluorescence (Figure 4A). Mitochondrial dysfunction in HepG2 cells following 24 h treatment with **3d** and **3k**, was also evident from the levels of intracellular ROS, revealed by cytofluorimetric analysis with 2′,7′-dichlorofluorescin diacetate, significantly higher than cell control (Figure 4B).

## 3. Experimental Section

### 3.1. Chemistry

#### 3.1.1. General

All melting points were taken on a Büchi-Tottoly capillary apparatus. IR spectra were determined in bromoform with a Shimadzu FT/IR 8400S spectrophotometer. ^1^H and ^13^C NMR spectra were measured at 200 and 50.0 MHz, respectively, in dimethylsulfoxide (DMSO)-*d_6_* or CDCl_3_ solution, using a Bruker Avance II series 200 MHz spectrometer. Compounds **3a**,**b** were characterized only by ^1^H NMR spectra because of their poor solubility. Column chromatography was performed with Merk silica gel 230–400 mesh ASTM or with Büchi Sepacor chromatography module (prepacked cartridge system). Elemental analyses (C, H, N) were within ±0.4% of theoretical values and were performed with a VARIO EL III elemental analyzer. Purity of all the tested compounds was greater than 98%, determined by HPLC as described below.

#### 3.1.2. Synthesis of 5-Fluoro-1-methyl-1*H*-indole (**7b**)

To a cold solution of 5-fluoroindole **7a** (0.8 g, 5.0 mmol) in anhydrous toluene (50 mL), potassium *t*-butoxide (0.8 g, 6.8 mmol) and tris[2-(2-methoxyethoxy)ethyl]amine (TDA-1) (1–2 drops) were added. The reaction mixture was stirred at room temperature for 5 h and then methyl iodide (0.3 mL, 5 mmol) was added. TLC analysis (dichloromethane (DCM)/petroleum ether (9/1) revealed that methylation was complete after 1 h. The solvent was evaporated under reduced pressure and the residue, treated with water (15 mL), was extracted with DCM (3 × 15 mL), dried (Na_2_SO_4_) and evaporated to afford the pure methyl derivative **7b**.

Yellow solid; yield: 98%; mp: 55–56 °C. Analytical and spectroscopic data were in agreement with those previously reported [50].

#### 3.1.3. Synthesis of 5-Fluoro-1-[(4-methylphenyl)sulfonyl]-1*H*-indole (**7c**)

To a stirred solution of 5-fluoroindole **7a** (1.0 g, 7.4 mmol) in tetrahydrofuran (THF) (5.0 mL) sodium hydride (60% dispersion in mineral oil, 0.4 g, 9.6 mmol) was added at 0 °C and the mixture was stirred at room temperature for 24 h. *p*-Toluenesulfonyl chloride (2.1 g, 11.1 mmol) was added to the reaction and the mixture was stirred at room temperature for 4 h. The residue was evaporated under reduced pressure, treated with water (20 mL) and extracted with ethyl acetate (EtOAc) (3 × 20 mL). The organic phase was dried (Na_2_SO_4_), evaporated under reduced pressure and purified by column chromatography using petroleum ether/DCM (9/1) as eluent. White solid; yield: 96%; mp: 111–112 °C; IR 1373, 1172 (SO_2_) cm^−1^; ^1^H NMR (200 MHz, DMSO-*d_6_*) δ: 2.32 (s, 3H, CH_3_), 6.82–6.84 (m, 1H, H-7), 7.19 (td, 1H, *J* = 2.6, 9.2, 11.0 Hz, H-6), 7.39 (d, 2H, *J* = 8.7 Hz, H-3′, H-5′), 7.45 (d, 1H, *J* = 2.6 Hz, H-3), 7.87 (d, 2H, *J* = 8.7 Hz, H-2′, H-6′), 7.88–7.97 (m, 2H, H-2, H-4); ^13^C NMR (50 MHz, DMSO-*d_6_*) δ: 21.0 (q), 107.0 (d, *J_C4_*_–*F*_ = 24.1 Hz), 109.3 (d, *J_C3_*_–*F*_ = 4.2 Hz), 112.5 (d, *J_C6_*_–*F*_ = 25.8 Hz), 114.4 (d, *J_C7_*_–*F*_ = 9.6 Hz), 126.7 (2xd), 128.9 (d), 130.2 (2xd), 130.6 (s), 131.6 (d, *J_C3a_*_–*F*_ = 10.4 Hz), 133.9 (s), 145.6 (s), 158.9 (d, *J_C5_*_–*F*_ = 237.7 Hz). Anal. Calcd for: C_15_H_12_FNO_2_S: C, 62.27; H, 4.18; N, 4.84. Found: C, 62.57; H, 4.38; N, 4.69.

#### 3.1.4. Synthesis of 1-(1-Methyl-1*H*-indol-3-yl)ethanone (**8b**)

To a cold solution of 3-acetylindole **8a** (1.0 g, 6.3 mmol) in anhydrous toluene (50 mL), potassium *t*-butoxide (1.0 g, 8.6 mmol) and TDA-1 (1–2 drops) were added. The reaction mixture was stirred at room temperature for 8 h and then methyl iodide (0.4 mL, 6.3 mmol) was added. TLC analysis (DCM/EtOAc 9/1) revealed that methylation was complete after 2 h. The solvent was evaporated under reduced pressure. The residue was treated with water (50 mL), extracted with DCM (3 × 50 mL), dried (Na_2_SO_4_), evaporated under reduced pressure, and purified by column chromatography using DCM/EtOAc (9/1) as eluent.

White solid; yield 80%; mp: 108–109 °C. Analytical and spectroscopic data were in agreement with those previously reported [48].

#### 3.1.5. Synthesis of 1-{1-[(4-Methylphenyl)sulfonyl]-1*H*-indol-3-yl}ethanone (**8c**)

To a solution of 3-acetylindole **8a** (1.0 g, 6.3 mmol) in THF (5.0 mL) sodium hydride (60% dispersion in mineral oil, 0.3 g, 6.3 mmol) was added at 0 °C and the mixture was stirred at room temperature for 1 h. *p*-toluenesulfonyl chloride (1.2 g, 6.3 mmol) was added and the mixture was stirred at room temperature for 24 h. The residue was evaporated under reduced pressure, treated with water (20 mL) and extracted with EtOAc (3 × 20 mL). The organic phase was dried (Na_2_SO_4_), evaporated under reduced pressure and purified by column chromatography using DCM as eluent. White solid; yield: 90%; mp: 148–149 °C; IR 1662 (CO), 1382, 1299 (SO_2_) cm^−1^; ^1^H NMR (200 MHz, DMSO-*d_6_*) δ: 2.33 (s, 3H, CH_3_), 2.60 (s, 3H, CH_3_), 7.32–7.39 (m, 2H, H-5, H-6), 7.43 (d, 2H, *J* = 7.8 Hz, H-3′, H-5′), 7.95 (d, 1H, *J* = 7.5 Hz, H-7), 8.04 (d, 2H, *J* = 7.8 Hz, H-2′, H-6′), 8.19 (d, 1H, *J* = 7.5 Hz, H-4), 8.81 (s, 1H, H-2); ^13^C NMR (50 MHz, DMSO-*d_6_*) δ:21.0 (q), 27.8 (q), 113.0 (d), 120.7 (s), 122.3 (d), 124.8 (d), 125.6 (d), 127.0 (s), 127.2 (2xd), 130.5 (2xd), 133.5 (s), 134.0 (s), 134.2 (d), 146.2 (s), 193.9 (s). Anal. Calcd for: C_17_H_15_NO_3_S: C, 65.16; H, 4.82; N, 4.47. Found: C, 65.28, H, 5.06, N, 4.37.

#### 3.1.6. Synthesis of 2-Chloro-1-(5-fluoro-1*H*-indol-3-yl)ethanones (**9a**,**b**)

To a stirred suspension of anhydrous aluminium chloride (1.1 g, 8.5 mmol), in DCM (18.0 mL), was added dropwise at 0 °C a solution of the suitable indole **7b**,**c** (1.2 mmol) in DCM (5.0 mL) under nitrogen atmosphere. Then, chloroacetyl chloride (0.3 mL, 3.6 mmol) was slowly added to the reaction mixture, which was stirred at room temperature for 1 h and then poured in ice and water (20 mL) and extracted with DCM (3 × 20 mL). The organic phase was dried (Na_2_SO_4_), evaporated under reduced pressure and purified by column chromatography using DCM as eluent.

##### 3.1.6.1. 2-Chloro-1-(5-fluoro-1-methyl-1*H*-indol-3-yl)ethanone (**9a**)

White solid; yield: 50%; mp: 185–186 °C; IR 1653 (CO) cm^−1^; ^1^H NMR (200 MHz, DMSO-*d_6_*) δ: 3.89 (s, 3H, CH_3_), 4.84 (s, 2H, CH_2_), 7.20 (td, 1H, *J* = 2.6, 9.2, 11.0 Hz, H-6), 7.62 (dd, 1H, *J* = 4.5, 9.2 Hz, H-7), 7.84 (dd, 1H, *J* = 2.6, 11.0 Hz, H-4), 8.51 (bs, 1H, H-2);^13^C NMR (50 MHz, DMSO-*d_6_*) δ: 33.7 (q), 46.2 (t), 106.1 (d, *J_C4–F_*= 24.8 Hz), 111.3 (d, *J_C6–F_* = 26.1 Hz), 112.3 (s), 112.4 (d, *J_C7–F_* = 9.8 Hz), 126.4 (d, *J_C3a–F_* = 11.0 Hz), 133.9 (s), 139.5 (d), 159.0 (d, *J_C5–F_* = 236 Hz), 185.6 (s). Anal. Calcd for C_11_H_9_ClFNO: C, 58.55; H, 4.02; N, 6.21. Found: C, 58.85; H, 4.14; N, 6.01.

##### 3.1.6.2. 2-Chloro-1-{5-fluoro-1-[(4-methylphenyl)sulfonyl]-1*H*-indol-3-yl}ethanone (**9b**)

White solid; yield: 55%; mp: 155–156 °C; IR 1683 (CO), 1375, 1171 (SO_2_) cm^−1^; ^1^H NMR (200 MHz, CDCl_3_) δ: 2.39 (s, 3H, CH_3_), 4.54 (s, 2H, CH_2_), 7.13 (td, 1H, *J* = 2.6, 8.9, 11.0 Hz, H-6), 7.31 (d, 2H, *J* = 8.0 Hz, H-3′, H-5′), 7.82 (d, 2H, *J* = 8.0 Hz, H-2′, H-6′), 7.90 (dd, 1H, *J* = 4.9, 8.9 Hz, H-7), 7.98 (dd, 2H, *J* = 2.6, 11.0 Hz, H-4), 8.34 (s, 1H, H-2); ^13^C NMR (50 MHz, CDCl_3_) δ: 21.7 (q), 45.8 (t), 108.9 (d, *J_C6–F_* = 25.5 Hz), 114.1 (s), 114.2 (d), 114.5 (d, *J_C4–F_* = 14.0 Hz), 117.9 (s, *J_C7a–F_* = 4.5 Hz), 127.2 (2xd), 130.2 (s), 130.4 (2xd), 133.5 (d), 134.1 (s), 146.5 (s), 160.1 (s, *J_C5–F_* = 241 Hz), 186.7 (s). Anal. Calcd for C_17_H_13_ClFNO_3_S: C, 55.82; H, 3.58; N, 3.83. Found: C, 55.99; H, 3.36; N, 4.10.

#### 3.1.7. Synthesis of 2-Bromo-(1*H*-indol-3-yl)ethanones (**9c**,**d**)

To a cold suspension of the appropriate 3-acetylindole **8b**,**c** (1.9 mmol) in anhydrous methanol (3.0 mL) bromine (0.1 mL, 1.9 mmol) was added dropwise. The mixture was heated at reflux for 2 h. After cooling the solvent was evaporated under reduced pressure. The residue was treated with water (20 mL), made alkaline by adding sodium hydrogen carbonate (150 mg) and extracted with EtOAc (3 × 50 mL). The organic phase was dried (Na_2_SO_4_), evaporated under reduced pressure and purified by column chromatography using DCM as eluent.

##### 3.1.7.1. 2-Bromo-1-(1-methyl-1*H*-indol-3-yl)ethanone (**9c**)

Brown solid; yield: 70%; mp: 205–206 °C. Analytical and spectroscopic data were in agreement with those previously reported [48].

##### 3.1.7.2. 2-Bromo-1-{1-[(4-methylphenyl)sulfonyl]-1*H*-indol-3-yl}ethanone (**9d**)

Brown solid; yield: 40%; mp: 134–135 °C; IR 1668 (CO), 1379, 1177 (SO_2_) cm^−1^; ^1^H NMR (200 MHz, CDCl_3_) δ: 2.38 (s, 3H, CH_3_), 4.36 (s, 2H, CH_2_), 7.29 (d, 2H, *J* = 8.2 Hz, H-3′, H-5′), 7.36–7.40 (m, 2H, H-5, H-6), 7.83–7.87 (m, 2H, H-2′, H-6′), 7.91–7.96 (m, 1H, H-7), 8.28–8.34 (m, 2H, H-2, H-4); ^13^C NMR (50 MHz, CDCl_3_) δ: 21.7 (q), 31.4 (t), 113.1 (d), 123.1 (d), 124.3 (s), 125.1 (d), 126.1 (d), 127.2 (2xd), 127.5 (s), 130.4 (2xd), 132.8 (d), 134.2 (s), 134.8 (s), 146.2 (s), 187.0 (s). Anal. Calcd. for: C_17_H_14_BrNO_3_S: C, 52.05; H, 3.60; N, 3.57. Found: C, 52.25; H, 3.70; N, 3.35.

#### 3.1.8. Synthesis of 3-[4-(1*H*-indol-3-yl)-1,3-thiazol-2-yl]-1*H*-pyrrolo[2,3-*b*]pyridines (**3a**–**h**)

A suspension of the appropriate pyrrolo[2,3-*b*]pyridine-carbothioamide **6a**,**b** (5.0 mmol) and halo-acetyl compounds **9a**–**d** (5.0 mmol) in ethanol (3.0 mL) was heated under reflux for 30 min. The precipitate, obtained after cooling, was filtered off, dried and recrystallized from ethanol to afford derivatives **3a**–**h**.

##### 3.1.8.1. 3-[4-(5-Fluoro-1-methyl-1*H*-indol-3-yl)-1,3-thiazol-2-yl]-1*H*-pyrrolo[2,3-*b*]pyridine (**3a**)

Yellow solid; yield: 97%; mp: 237–238 °C; IR 3550 (NH) cm^−1^; ^1^H NMR (200 MHz, DMSO-*d_6_*) δ: 3.90 (s, 3H, CH_3_), 7.12 (t, 1H, *J* = 8.2 Hz, H-6″), 7.44 (dd, 1H, *J* = 5.2, 7.4 Hz, H-5′), 7.54 (dd, 1H, *J* = 4.6, 8.2 Hz, H-7″), 7.73 (s, 1H, H-2″), 7.94 (dd, 1H, *J* = 9.7, 2.5 Hz, H-4″), 8.16 (bs, 1H, H-2′), 8.38 (s, 1H, H-5), 8.45 (d, 1H, *J* = 3.7 Hz, H-6′), 8.89 (d, 1H, *J* = 7.4 Hz, H-4′), 12.74 (bs, 1H, NH). Anal. Calcd for C_19_H_13_FN_4_S: C, 65.50; H, 3.76; N, 16.08. Found: C, 65.76; H, 3.55; N, 16.22.

##### 3.1.8.2. 3-[4-(5-Fluoro-1-methyl-1*H*-indol-3-yl)-1,3-thiazol-2-yl]-1-methyl-1*H*-pyrrolo[2,3-*b*]pyridine (**3b**)

Yellow solid; yield: 30%; mp: 286–287 °C; ^1^H NMR (200 MHz, DMSO-*d_6_*) δ: 2.56 (s, 3H, CH_3_), 4.03 (s, 3H, CH_3_), 6.02 (dd, 1H, *J* = 4.3, 10.7 Hz, H-7″), 6.76 (td, 1H, *J* = 2.3, 9.5, 10.7 Hz, H-6″), 6.98 (dd, 1H, *J* = 5.0, 8.4 Hz, H-5′), 7.25 (dd, 1H, *J* = 2.3, 9.5 Hz, H-4″), 7.72 (d, 1H, *J* = 8.4 Hz, H-4′), 8.19-8.81 (m, 4H, H-2’, H-2’’, H-5, H-6′). Anal. Calcd for C_20_H_15_FN_4_S: C, 66.28; H, 4.17; N, 15.46. Found: C, 66.53; H, 3.90; N, 15.23.

##### 3.1.8.3. 3-[4-(1-Methyl-1*H*-indol-3-yl)-1,3-thiazol-2-yl]-1*H*-pyrrolo[2,3-*b*]pyridine (**3c**)

Yellow solid; yield: 60%; mp: 274–275 °C, IR 3427 (NH) cm^−1^; ^1^H NMR (200 MHz, DMSO-*d_6_*) δ: 3.90 (s, 3H, CH_3_), 7.21–7.30 (m, 2H, H-5″, H-6″), 7.36 (dd, 1H, *J* = 4.9, 7.7 Hz, H-5′), 7.53 (d, 1H, *J* = 7.1 Hz, H-7″), 7.66 (s, 1H, H-2″), 8.05 (s, 1H, H-5), 8.19 (d, 1H, *J* = 7.1 Hz, H-4″), 8.30 (d, 1H, *J* = 2.0 Hz, H-2′), 8.40 (d, 1H, *J* = 4.9 Hz, H-6′), 8.79 (d, 1H, *J* = 7.7 Hz, H-4′), 12.40 (bs, 1H, NH); ^13^C NMR (50 MHz, DMSO-*d_6_*) δ: 32.6 (q), 106.5 (d), 109.6 (s), 110.2 (d), 117.1 (d), 117.3 (s), 119.9 (d), 120.1 (d), 121.6 (d), 124.9 (s), 126.9 (d), 129.2 (d), 129.7 (d), 137.1 (s), 143.2 (d), 143.3 (s), 147.9 (s), 150.1 (s), 161.0 (s). Anal. Calcd for C_19_H_14_N_4_S: C, 69.07; H, 4.27; N, 16.96. Found: C, 68.90; H, 4.46; N, 16.75.

##### 3.1.8.4. 1-Methyl-3-[4-(1-methyl-1*H*-indol-3-yl)-1,3-thiazol-2-yl]-1*H*-pyrrolo[2,3-*b*]pyridine (**3d**)

Yellow solid; yield: 60%; mp: 290–291 °C; ^1^H NMR (200 MHz, DMSO-*d_6_*) δ: 3.90 (s, 3H, CH_3_), 3.94 (s, 3H, CH_3_), 7.17–7.30 (m, 2H, H-5″, H-6″), 7.39 (dd, 1H, *J* = 4.7, 7.9 Hz, H-5′), 7.53 (dd, 1H, *J* = 1.4, 6.9 Hz, H-7″), 7.66 (s, 1H, H-2″), 8.05 (s, 1H, H-2′), 8.19 (dd, 1H, *J* = 1.4, 6.9 Hz, H-4″), 8.41 (s, 1H, H-5), 8.45 (dd, 1H, *J* = 1.3, 4.7 Hz, H-6′), 8.78 (dd, 1H, *J* = 1.3, 7.9 Hz, H-4′); ^13^C NMR (50 MHz, DMSO-*d_6_*) δ: 31.4 (q), 32.6 (q), 106.5 (d), 108.3 (s), 110.0 (s), 110.2 (d), 117.2 (d), 117.5 (s), 119.9 (d), 120.1 (d), 121.6 (d), 124.9 (s), 129.3 (d), 129.8 (d), 130.5 (d), 137.0 (s), 143.1 (d), 147.0 (s), 150.1 (s), 160.7 (s). Anal. Calcd for C_20_H_16_N_4_S: C, 69.74; H, 4.68; N, 16.27. Found: C, 69.50; H, 4.84; N, 16.06.

##### 3.1.8.5. 3-(4-{5-Fluoro-1-[(4-methylphenyl)sulfonyl]-1*H*-indol-3-yl}-1,3-thiazol-2-yl)-1*H*-pyrrolo [2,3-*b*]pyridine (**3e**)

Yellow solid; yield: 97%; mp: 257–258 °C; IR 3337 (NH), 1370, 1171 (SO_2_); ^1^H NMR (200 MHz, DMSO-*d_6_*) δ: 2.31 (s, 3H, CH_3_), 7.28–7.32 (m, 2H, ArH), 7.39–7.42 (m, 2H, H-3‴, H-5‴), 7.94–7.98 (m, 2H, H-2‴, H-6‴), 8.03–8.15 (m, 3H, ArH), 8.42–8.49 (m, 3H, ArH), 8.74 (d, 1H, *J* = 7.4 Hz, H-4′), 12.67 (s, 1H, NH); ^13^C NMR (50 MHz, DMSO-*d_6_*) δ: 21.1 (q), 107.3 (d, *J_C4″–F_* = 24.1 Hz), 109.3 (s), 112.6 (d, *J_C6″–F_* = 26.8 Hz), 113.4 (d), 114.9 (d, *J_C7″–F_* = 9.9 Hz), 117.2 (d), 117.4 (s), 117.5 (s), 117.6 (s), 126.3 (d), 126.9 (2xd), 127.8 (d), 128.8 (s), 129.0 (s), 130.2 (d), 130.4 (2xd), 131.1 (s), 133.6 (s), 142.3 (d), 145.9 (s), 146.9 (s), 159.4 (d, *J_C5″–F_* = 245.5 Hz). Anal. Calcd. for C_25_H_17_FN_4_O_2_S_2_: C, 61.46; H, 3.51; N, 11.47. Found: C, 61.73; H, 3.25; N, 11.31.

##### 3.1.8.6. 3-(4-{5-Fluoro-1-[(4-methylphenyl)sulfonyl]-1*H*-indol-3-yl}-1,3-thiazol-2-yl)-1-methyl-1*H*-pyrrolo[2,3-*b*]pyridine (**3f**)

White solid; yield: 40%; mp: 230–231 °C; IR 1380, 1299 (SO_2_) cm^−1^; ^1^H NMR (200 MHz, DMSO-*d_6_*) δ: 2.32 (s, 3H, CH_3_), 3.94 (s, 3H, CH_3_), 7.28–7.43 (m, 2H, ArH), 7.41 (d, 2H, *J* = 7.8 Hz, H-3‴, H-5‴), 7.87–7.94 (m, 2H, H-2‴, H-6‴), 7.99–8.13 (m, 3H, ArH), 8.42–8.48 (m, 3H, ArH), 8.60 (d, 1H, *J* = 7.3 Hz, H-4′); ^13^C NMR (50 MHz, DMSO-*d_6_*) δ: 21.0 (q), 31.2 (q), 107.4 (d, *J_C4″–F_* = 25.2 Hz), 107.8 (s), 112.0 (d), 113.1 (d, *J_C6″–F_* = 27.5 Hz), 114.8 (d, *J_C7″–F_* = 9.1 Hz), 116.9 (s), 117.4 (d), 117.5 (d, *J_C7″–a_* = 4.0 Hz), 126.3 (d), 126.9 (2xd), 128.6 (d), 128.8 (s), 129.0 (s), 130.4 (s), 130.8 (2xd), 131.1 (s), 133.6 (s), 143.8 (d), 145.9 (s), 146.9 (s), 147.6 (s), 159.3 (d, *J_C5″–F_* = 246.5 Hz). Anal. Calcd. for C_26_H_19_FN_4_O_2_S_2_: C, 62.13; H, 3.81; N, 11.15. Found: C, 61.98; H, 3.99; N, 10.90.

##### 3.1.8.7. 3-(4-{1-[(4-Methylphenyl)sulfonyl]-1*H*-indol-3-yl}-1,3-thiazol-2-yl)-1*H*-pyrrolo[2,3-*b*] pyridine (**3g**)

Yellow solid; yield: 90%; mp: 260–261 °C; IR 3498 (NH), 1374, 1174 (SO_2_) cm^−1^; ^1^H NMR (200 MHz, DMSO-*d_6_*) δ: 2.30 (s, 3H, CH_3_), 7.31–7.41 (m, 5H, ArH), 7.94–7.98 (m, 2H, ArH), 8.01–8.07 (m, 2H, ArH), 8.36–8.39 (m, 4H, ArH), 8.64 (d, 1H, *J* = 7.7 Hz, H-4′), 12.39 (bs, 1H, NH);^13^C NMR (50 MHz, DMSO-*d_6_*) δ: 21.0 (q), 109.1 (s), 111.8 (d), 113.4 (d), 116.7 (s), 117.2 (d), 117.7 (s), 121.7 (d), 124.0 (d), 124.5 (d), 125.2 (d), 126.8 (2xd), 127.2 (d), 127.9 (s), 128.6 (d), 130.3 (2xd), 133.8 (s), 134.6 (s), 144.0 (d), 145.7 (s), 147.3 (s), 148.7 (s), 162.1 (s). Anal. Calcd. for C_25_H_18_N_4_O_2_S_2_: C, 63.81; H, 3.86; N, 11.91. Found: C, 63.55; H, 4.02; N, 11.76.

##### 3.1.8.8. 1-Methyl-3-(4-{1-[(4-methylphenyl)sulfonyl]-1*H*-indol-3-yl}-1,3-thiazol-2-yl)-1*H*-pyrrolo [2,3-*b*]pyridine (**3h**)

Yellow solid; yield: 50%; mp: 250–251 °C; IR 1374, 1174 (SO_2_) cm^−1^; ^1^H NMR (200 MHz, DMSO-*d_6_*) δ: 2.30 (s, 3H, CH_3_), 3.94 (s, 3H, CH_3_), 7.37–7.45 (m, 5H, ArH), 7.93–7.97 (m, 2H, ArH), 8.01–8.09 (m, 2H, ArH), 8.31–8.47 (m, 4H, ArH), 8.66 (d, 1H, *J* = 7.5 Hz, H-4′);^13^C NMR (50 MHz, DMSO-*d_6_*) δ: 21.0 (q), 31.4 (q), 108.0 (s), 112.0 (d), 113.4 (d), 117.2 (s), 117.4 (d), 117.6 (s), 121.7 (d), 124.0 (d), 124.6 (d), 125.2 (d), 126.8 (2xd), 127.8 (s), 129.3 (d), 130.3 (2xd), 130.9 (d), 133.8 (s), 134.6 (s), 145.7 (s), 147.1 (s), 147.4 (s), 148.2 (d), 161.6 (s). Anal. Calcd for: C_26_H_20_N_4_O_2_S_2_: C, 64.44; H, 4.16, N, 11.56. Found: C, 64.19; H, 4.33; N, 11.25.

#### 3.1.9. Synthesis of 3-[4-(1*H*-indol-3-yl)-1,3-thiazol-2-yl]-1*H*-pyrrolo[2,3-*b*]pyridines (**3i**–**k**)

To a suspension of derivatives **3e**–**h** (0.6 mmol) in ethanol (2 mL), potassium hydroxide (0.1 g, 1.8 mmol) was added and the mixture was heated at reflux for 1–2 h. The precipitate, obtained after cooling, was filtered off, dried and recrystallized from ethanol to afford derivatives **3i**–**k**.

##### 3.1.9.1. 3-[4-(5-Fluoro-1*H*-indol-3-yl)-1,3-thiazol-2-yl]-1*H*-pyrrolo[2,3-*b*]pyridine (**3i**)

Conditions: reflux for 1.5 h. Yellow solid; yield: 98%; mp: 248–249 °C; IR 3600 and 3550(2xNH), cm^−1^; ^1^H NMR (200 MHz, DMSO-*d_6_*) δ: 7.05 (t, 1H, *J* = 7.8 Hz, H-6″), 7.47–7.53 (m, 2H, H-5′, H-7″), 7.78 (bs, 1H, H-2″), 7.92 (d, 1H, *J* = 8.9 Hz, H-4″), 8.19 (bs, 1H, H-2′), 8.47 (m, 2H, H-5, H-6′), 8.95 (d, 1H, *J* = 7.4 Hz, H-4′), 11.70 (bs, 1H, NH), 12.93 (bs, 1H, NH); ^13^C NMR (50 MHz, DMSO-*d_6_*) δ: 104.7 (d, *J_C4″–F_* = 21.6 Hz), 107.3 (d), 109.7 (d, *J_C6″–F_* = 26.1 Hz), 109.8 (s), 110.7 (s), 113.0 (d, *J_C7″–F_* = 10.0 Hz), 117.2 (d), 119.0 (s), 124.6 (d, *J_C3″–F_* = 10.5 Hz), 127.2 (d), 128.2 (d), 132.3 (d), 133.3 (s), 140.6 (d), 144.7 (s), 149.6 (s), 157.5 (d, *J_C5″–F_* = 232 Hz), 160.5 (s). Anal. Calcd. for C_18_H_11_FN_4_S: C, 64.66; H, 3.32; N, 16.76. Found: C, 64.37; H, 3.04; N, 16.57.

##### 3.1.9.2. 3-[4-(5-Fluoro-1*H*-indol-3-yl)-1,3-thiazol-2-yl]-1-methyl-1*H*-pyrrolo[2,3-*b*]pyridine (**3l**)

Conditions: reflux for 1 h. Yellow solid; yield 40%; mp: 243–244 °C; IR 3557 (NH) cm^−1^; ^1^H NMR (200 MHz, DMSO-*d_6_*) δ: 3.93 (s, 3H, CH_3_), 7.04 (td, 1H, *J* = 2.1, 9.1, 10.9 Hz, H-6″), 7.35 (dd, 1H, *J* = 4.6, 7.8 Hz, H-5′), 7.49 (dd, 1H, *J* = 4.6, 9.1 Hz, H-7″), 7.70 (s, 1H, H-2″), 7.96 (dd, 1H, *J* = 2.1, 10.9 Hz, H-4″), 8.11 (bs, 1H, H-2′), 8.38 (s, 1H, H-5), 8.42 (d, 1H, *J* = 4.6 Hz, H-6′), 8.69 (d, 1H, *J* = 7.8 Hz, H-4′), 11.64 (bs, 1H, NH);^13^C NMR (50 MHz, DMSO-*d_6_*) δ: 31.2 (q), 104.9 (d, *J_C4″–F_* = 24 Hz), 106.5 (d), 108.3 (s), 109.7 (d, *J_C6″–F_* = 26 Hz), 111.3 (d, *J_C7″–F_* = 4.5 Hz), 112.9 (d, *J_C7″–F_* = 9.5 Hz), 117.0 (s), 117.2 (d), 124.8 (d, *J_C4″–F_* = 10.1 Hz), 126.8 (d), 129.0 (d), 130.2 (d), 133.3 (s), 143.7 (d), 147.6 (s), 150.2 (s), 157.4 (d, *J_C5″–F_* = 231.9 Hz), 160.8 (s). Anal. Calcd. for C_19_H_13_FN_4_S: C, 65.50; H, 3.76; N, 16.08. Found: C, 65.36; H, 3.47; N, 15.82.

##### 3.1.9.3. 3-[4-(1*H*-Indol-3-yl)-1,3-thiazol-2-yl]-1*H*-pyrrolo[2,3-*b*]pyridine (**3j**)

Conditions: reflux for 2 h. Yellow solid; yield: 98%; mp: 297–298 °C; IR 3676 and 3557 (2xNH) cm^−1^; ^1^H NMR (200 MHz, DMSO-*d_6_*) δ: 7.13–7.23 (m, 2H, H-5″, H-6″), 7.39 (dd, 1H, *J* = 4.9, 7.9 Hz, H-5′), 7.47–7.51 (m, 1H, H-7″), 7.68 (bs, 1H, H-2″), 8.05 (d, 1H, *J* = 2.6 Hz, H-2′), 8.18 (dd, 1H, *J* = 2.7, 7.1 Hz, H-4″), 8.34 (s, 1H, H-5), 8.42 (dd, 1H, *J* = 1.5, 4.9 Hz, H-6′), 8.82 (dd, 1H, *J* = 1.5, 7.9 Hz, H-4′), 11.46 (bs, 1H, NH), 12.50 (bs, 1H, NH); ^13^C NMR (50 MHz, DMSO-*d_6_*) δ: 106.6 (d), 109.7 (s), 110.9 (s), 111.9 (d), 117.1 (d), 117.6 (s), 119.8 (d), 120.0 (d), 121.5 (d), 124.6 (s), 125.1 (d), 127.1 (d), 130.3 (d), 136.6 (s), 142.7 (d), 147.3 (s), 150.5 (s), 160.8 (s). Anal. Calcd for C_18_H_12_N_4_S: C, 68.33; H, 3.82; N, 17.71. Found: C, 68.06; H, 4.07; N, 17.45.

##### 3.1.9.4. 3-[4-(1*H*-Indol-3-yl)-1,3-thiazol-2-yl]-1-methyl-1*H*-pyrrolo[2,3-*b*]pyridine (**3k**)

Conditions: reflux for 1.5 h. Yellow solid; yield: 98%; mp: 276–277 °C; IR 3500 (NH) cm^−1^; ^1^H NMR (200 MHz, DMSO-*d_6_*) δ: 3.95 (s, 3H, CH_3_), 7.14–7.25 (m, 2H, H-5″, H-6″), 7.41 (dd, 1H, *J* = 5.0, 7.7 Hz, H-5′), 7.50 (dd, 1H, *J* = 2.1, 7.4 Hz, H-7″), 7.69 (bs, 1H, H-2″), 8.05 (d, 1H, *J* = 5.0 Hz, H-6′), 8.16–8.21 (m, 1H, H-4″), 8.45–8.48 (m, 2H, H-5, H-2′), 8.78 (dd, 1H, *J* = 2.0, 7.7 Hz, H-4′), 11.49 (s, 1H, NH); ^13^C NMR (50 MHz, DMSO-*d_6_*) δ: 31.6 (q), 106.8 (d), 108.4 (s), 110.7 (s), 111.9 (d), 117.3 (d), 119.8 (d), 119.9 (d), 121.6 (d), 124.6 (s), 125.1 (d), 125.9 (s), 130.3 (d), 130.8 (d), 135.7 (s), 136.6 (s), 142.6 (d), 150.3 (s), 160.6 (s). Anal. Calcd for C_19_H_14_N_4_S: C, 69.07; H, 4.27; N, 16.96. Found: C, 69.31; H, 4.50; N, 16.70.

### 3.2. HPLC Analysis

Analysis of nortopsentin analogues was carried out using a Gilson modular liquid chromatography system (Gilson Inc., Middleton, WI, USA) equipped with M 302 and 305 pumps, and injector model 77–25 (Rheodyne, Berkeley, CA, USA) with a 20 μL injector loop and a M 802 manometric module. The chromatographic column was a µPorasil column (300 × 3.9 mm; Waters, Milford, MA, USA) provided with relevant guard cartridge (5 × 3.9 mm, Waters). Detection at 283 nm was by an M 118 UV-vis detector, used along with the Gilson 712 HPLC system controller software. Sensitivity was 0.05% absorbance unit (AUFS). Elution was with a 20 min linear gradient elution from solvent A (dichlomethane:ethyl acetate, 1:1) to 100% solvent B (ethyl acetate), at a flow rate of 1 mL/min. Retention times of **3d** and **3k** were 8.10 min and 8.33 min, respectively.

### 3.3. Biology

#### 3.3.1. Viability Assay *in Vitro*

The tested compounds **3d** and **3k** were dissolved in DMSO and then diluted in culture medium so that the effective DMSO concentration did not exceed 0.1%. Human cell lines of hepatoma HepG2 and Chang liver were purchased from American Type Culture Collection, Rockville, MD, USA. Cells were grown in RPMI supplemented with 2 mM l-glutamine, 10% FBS, 100 U/mL penicillin, 100 μg/mL streptomycin and 5 μg/mL gentamicin. HepG2 culture medium also contained 1.0 mM sodium pyruvate. Cells were maintained in log phase by seeding twice a week at a density of 3 × 10^8^ cells/L in humidified 5% CO_2_ atmosphere, at 37 °C. In all experiments, cells were made quiescent through overnight incubation before the treatment with the compounds or vehicle alone (control cells). No differences were found between cells treated with DMSO 0.1% and untreated cells in terms of cell number and viability. Cytotoxic activity of the compounds was determined by the MTT quantitative colorimetric assay based on the reduction of the 3-(4,5-dimethyl-2-thiazolyl)-2,5-diphenyl-2*H*-tetrazolium bromide (MTT) into purple formazan by mitochondrial dehydrogenases of living cells. Briefly, HepG2 and Chang cells were plated at 5 × 10^4^ cells/well in 96-well plates containing 200 μL RPMI. After an overnight incubation, cells were washed with fresh medium and incubated with the compounds in RPMI. After a 72 h incubation, cells were washed, and 50 µL FBS-free medium containing 5 mg/mL MTT were added. The medium was discarded after 4 h incubation at 37 °C, and formazan blue formed in the cells was dissolved in DMSO. The absorbance at 540 nm was measured in a microplate reader (Bio-RAD, Hercules, CA, USA). The growth inhibition activity of compounds was defined as GI_50_value, which represents the log of the molar concentration of the compound that inhibits 50% cell growth.

#### 3.3.2. Cell Cycle Analysis

Cell cycle stage was analyzed by flow cytometry. Aliquots of 1 × 10^6^ cells were harvested by centrifugation, washed with PBS and incubated in the dark in a PBS solution containing 20 μg/mL propidium iodide (PI) and 200 μg/mL RNase, for 30 min, at room temperature. Then samples were subjected to fluorescence-activated cell sorting (FACS) analysis by Epics XL™ flow cytometer using Expo32 software (Beckman Coulter, Fullerton, CA, USA). At least 1 × 10^4^ cells were analyzed for each sample.

#### 3.3.3. Measurement of Phosphatidylserine Exposure

The apoptosis-induced PS externalization to the cell surface was measured by flow cytometry by double staining with Annexin V-Fluorescein isothiocyanate (Annexin V-FITC)/propidium iodide (PI). Annexin V binding to phosphatidylserine is used to identify the earliest stage of apoptosis. PI, which does not enter cells with intact membranes, is used to distinguish between early apoptotic cells (annexin V-FITC positive and PI negative), late apoptotic cells (annexin V-FITC/PI-double positive) or necrotic cells (annexin V-FITC negative and PI positive). HepG2 cells were seeded in triplicate in 24-wells culture plates at a density of 2.0 × 10^5^ cells/cm^2^. After an overnight incubation, cells were washed with fresh medium and incubated with the compounds in RPMI. After 24 h, cells were harvested by trypsinization and adjusted at 2.0 × 10^5^ cells/mL with combining buffer. One hundred μL of cell suspended solution was added to a new tube, and incubated with 5 µL annexin V and 10 μL of a 20 μg/mL PI solution at room temperature in the dark for 15 min. Then at least 1.0 × 10^4^ cells were immediately subjected to FACS analysis using appropriate 2-bidimensional gating method.

#### 3.3.4. Acridine Orange and Ethidium Bromide Morphological Fluorescence Dye Staining

Acridine orange (AO) stains DNA bright green, allowing visualization of the nuclear chromatin pattern and stains both live and dead cells. Ethidium bromide (EB) stains DNA orange but is excluded by viable cells. Dual staining allows separate enumeration of populations of viable non-apoptotic, viable (early) apoptotic, nonviable (late) apoptotic, and necrotic cells. Live cells appear uniformly green. Early apoptotic cells stain green and contain bright green dots in the nuclei as a consequence of chromatin condensation and nuclear fragmentation. Late apoptotic cells incorporate EB and therefore stain orange, but, in contrast to necrotic cells, the late apoptotic cells show condensed and often fragmented nuclei. Necrotic cells stain orange, but have a nuclear morphology resembling that of viable cells, with no condensed chromatin. Briefly, after HepG2 cells were treated with **3d** or **3k** compounds for 24 h, the medium was discarded. Cells were washed with saline 5 mM phosphate buffer, pH 7.4 (PBS) and then incubated with 100 μL PBS containing 100 μg/mL of EB plus 100 μg/mL of AO. After 20 s, EB/AO solution was discarded and cells immediately visualized by means of fluorescent microscope equipped with an automatic photomicrograph system (Leica, Wetzlar, Germany). Multiple photos were taken at randomly selected areas of the well to ensure that the data obtained are representative.

#### 3.3.5. Measurement of Mitochondrial Transmembrane Potential

Mitochondrial transmembrane potential was assayed by flow cytofluorometry, using the cationic lipophilic dye 3,30-dihexyloxacarbocyanine iodide (Molecular Probes, Inc., Eugene, OR, USA), which accumulates in the mitochondrial matrix. Changes in mitochondrial membrane potential are indicated by a reduction in the 3,30-dihexyloxacarbocyanine iodide-induced fluorescence intensity. Cells were incubated with 3,30-dihexyloxacarbocyanine iodide at a 40 nmol/L final concentration, for 15 min at 37 °C. After centrifugation, cells were washed with PBS and suspended in 500 µL PBS. Fluorescent intensities were analyzed in at least 1 × 10^4^ cells for each sample.

#### 3.3.6. Measurement of Intracellular Reactive Oxygen Species

ROS level was monitored by measuring fluorescence changes that resulted from intracellular oxidation of 2′,7′-dichlorofluorescin diacetate (DCFH). DCFH, at 10 mM final concentration, was added to the cell medium 30 min before the end of the treatment. The cells were collected by centrifugation for 5 min at 2000 rpm at 4 °C, washed, suspended in PBS and immediately subjected to fluorescence-activated cell sorting analysis. At least 1 × 10^4^ cells were analyzed for each sample.

## 4. Conclusions

A new series of nortopsentin analogues in which the imidazole ring of the natural product was replaced by thiazole and the indole unit bound to the position 2 of thiazole was substituted by a 7-azaindole moiety was efficiently synthesized. Two of the new nortopsentin derivatives, **3d** and **3k**, showed good antiproliferative activity against the totality of the about 60 human tumor cell lines of NCI full panel with GI_50_ values ranging from low micromolar to nanomolar level (13.0–0.03 and 14.2–0.04 μM, respectively. Moreover, they have shown potent cytotoxic activity on HepG2 hepatocarcinoma cells, while under identical conditions, they did not affect normal immortalized human liver cells (Chang). Both the compounds induced a concentration-dependent accumulation of cells in the subG0/G1phase while confined viable cells in G2/M phase. The mechanism of the anti-proliferative effect of the nortopsentin derivatives was pro-apoptotic, being associated with externalization of plasma membrane phosphatidylserine and mitochondrial dysfunction.
